# Transapical beating-heart septal myectomy for hypertrophic cardiomyopathy with latent obstruction

**DOI:** 10.1093/ejcts/ezad425

**Published:** 2023-12-19

**Authors:** Jiangtao Li, Xiang Wei

**Affiliations:** Division of Cardiovascular Surgery, Tongji Hospital, Tongji Medical College, Huazhong University of Science and Technology, Wuhan, China; Key Laboratory of Organ Transplantation, Ministry of Education, Wuhan, China; NHC Key Laboratory of Organ Transplantation, Ministry of Health, Wuhan, China; Division of Cardiovascular Surgery, Tongji Hospital, Tongji Medical College, Huazhong University of Science and Technology, Wuhan, China; Key Laboratory of Organ Transplantation, Ministry of Education, Wuhan, China; NHC Key Laboratory of Organ Transplantation, Ministry of Health, Wuhan, China

**Keywords:** Latent obstruction, Hypertrophic cardiomyopathy, Transapical beating-heart septal myectomy

## Abstract

**OBJECTIVES:**

A novel transapical beating-heart septal myectomy (TA-BSM) procedure was performed for patients with latent obstruction through the left intercostal incision and without cardiopulmonary bypass. This study aims to demonstrate the experience of the TA-BSM procedure for patients with latent obstruction and compare outcomes to patients with resting obstruction.

**METHODS:**

We studied 120 symptomatic hypertrophic obstructive cardiomyopathy patients (33 with latent obstruction and 87 with resting obstruction) who underwent TA-BSM. Demographic profiles, echocardiogram-derived ventricular morphology and haemodynamics and clinical outcomes were analysed.

**RESULTS:**

There were no important differences in baseline clinical characteristics between patients with latent obstruction and resting obstruction, including age, symptoms, comorbidities and medical history. Patients with latent obstruction had lower basal septum thickness, higher midventricular wall thickness, smaller left atrial chamber size and more frequency of mitral subvalvular anomalies. There was no difference in early (<30 days) deaths (0/33 vs 1/87, *P* > 0.999) and mid-term survival between patients with latent obstruction and resting obstruction. At 6 months after surgery, 31 (93.9%) patients with latent obstruction and 80 (92.0%) with resting obstruction achieved optimal procedural success, which was defined as a maximal gradient (after provocation) <30 mmHg and mitral regurgitation ≤ grade 1+ without mortality. Maximal left ventricular outflow tract gradient, basal septum thickness, midventricular wall thickness, mitral regurgitation grade and left atrial chamber size were significantly decreased after TA-BSM. In the follow-up, the New York Heart Association class was significantly improved following surgery.

**CONCLUSIONS:**

TA-BSM preserved favourable gold-standard guideline desired outcomes through real-time echocardiographic-guided resection. Equipoise of outcomes for this procedure regardless of degree of resting left ventricular outflow tract gradients supports operative management with this approach in symptomatic patients with latent obstruction.

**Figure:**
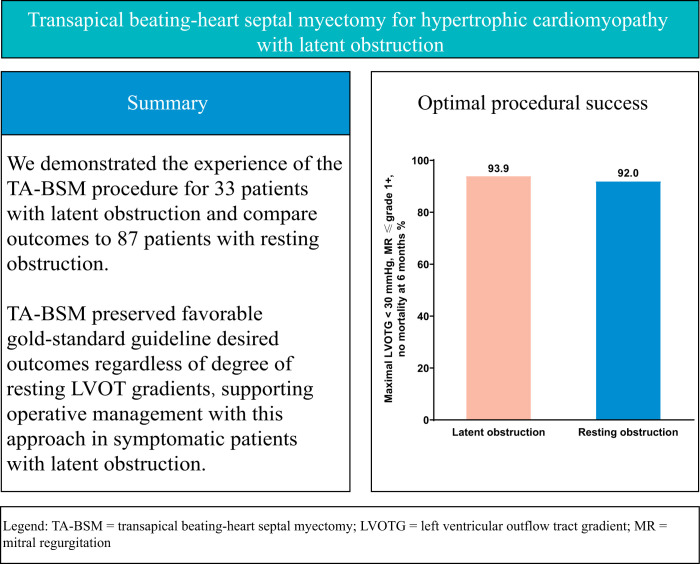

## INTRODUCTION

Hypertrophic cardiomyopathy (HCM) is the most common inherited cardiac disease reported in populations globally and has a prevalence of 1:500 in individuals [1]. In the majority of patients, the left ventricular (LV) septum is abnormally thickened, which leads to systolic anterior motion of the mitral valve, obstruction of the left ventricular outflow tract (LVOT) and mitral regurgitation (MR) [2]. In some patients, the LVOT gradient is minimal at rest but may increase dramatically after provocation [3]. Latent obstruction is considered as a subtype of obstructive HCM when there is no significant LVOT gradient at rest but increased ≥30 mmHg after provocation [4]. Latent obstruction can cause serious heart failure symptoms and is associated with unfavourable prognosis in HCM patients [4].

Septal myectomy (SM) has been recommended as the gold standard for patients with HCM and LVOT gradient >50 mmHg (at rest or with provocation), who remain severely symptomatic despite guideline-directed management and therapy [5, 6]. Our previous study has shown that, enabled by an innovative beating-heart myectomy device (BMD), the novel minimally invasive transapical beating-heart septal myectomy (TA-BSM) procedure achieved favourable results for patients with obstructive HCM, without median sternotomy and cardiopulmonary bypass (CPB) [7]. However, several studies reported that patients with latent obstruction had a higher prevalence of mitral subvalvular abnormalities and needed concomitant mitral subvalvular management [8, 9]. Therefore, it is unclear whether the TA-BSM procedure is also appropriate for patients with latent obstruction. This study aims to demonstrate our experience of the TA-BSM procedure for patients with latent obstruction and compare outcomes to patients with resting LVOT obstruction.

## PATIENTS AND METHODS

### Ethics statement

This study complied with the ethical standards of the Declaration of Helsinki and was approved by the Ethics Committee of the Tongji Medical College (approval number: 2022-S013, 2022-S013-1, 2022-S013-2, 2022-S013-3, 2022-149 S013-4). Patients consented to participate in this study and signed an informed consent form.

### Study population

Between April 2022 and February 2023, we conducted a single-centre, single-arm, first-in-man, clinical study on the TA-BSM procedure, which was performed by a single surgeon at Tongji Hospital (Wuhan, China). We prospectively collected data on HCM patients with latent or resting obstruction. Patients from all over China agreed to participate in this clinical trial after receiving comprehensive explanations regarding this procedure.

Inclusion criteria were as follows: patients with (i) a resting or provoked LVOT gradient > 50 mmHg and a maximal septal thickness ≥15 mm; (ii) drug refractory symptoms, including chest pain, dyspnoea or syncope/presyncope; and (iii) a consent form to approve the TA-BSM procedure. Patients were excluded if they were (i) pregnant; (ii) presented with severe heart failure with an LV ejection fraction <40%; and (iii) diagnosed with concomitant diseases needing open-heart surgery, such as primary valvular disease or coronary artery disease. Therefore, a total of 120 patients were included in this study.

### Preoperative echocardiography

Transthoracic echocardiography (TTE) was performed at baseline according to the recommendations of the American Society of Echocardiography [10]. The diagnostic criteria of HCM included a maximal wall thickness of ≥15 mm anywhere in the left ventricle and the absence of another cause of hypertrophy in adults. Resting obstruction was defined as an LVOT gradient ≥30 mmHg at rest. Patients with a resting LVOT gradient <30 mmHg were subjected to provocative manoeuvres using the Valsalva manoeuvre, treadmill test [11], isoproterenol infusion (0.01–0.1 μg/kg/min) or repetitive squat-to-stand manoeuvre [12]. The MR grades were 0 none, 1+ mild, 2+ moderate, 3+ moderate to severe and 4+ severe [13]. Assessments of LV diastolic function performed were the ratio between early mitral inflow velocity and mitral annular early diastolic velocity (*E*/*e′*) and left atria diameter. Apical outpouching was defined as persistent apical cavity in which the apical cavity dimension at end-systole was greater than in the midventricular cavity dimension at end-systole [14]. Apical aneurysm was defined as a discrete, thin-walled dyskinetic or akinetic segment of the most distal portion of the chamber, with a relatively wide communication to the LV cavity.

### Study protocol and follow‐up

All patients were stable and receiving optimal medical therapy at our centre. Patients were divided into 2 groups: the latent obstruction group (patients with provoked obstruction despite a low resting gradient of <30 mmHg) and the resting obstruction group (patients with resting LVOT obstruction, resting gradient of ≥30 mmHg). Baseline characteristics and perioperative results were obtained from the electronic database of our institution and were carefully reviewed. All patients received a cranial computed tomography (*n* = 65) or magnetic resonance imaging (MRI) (*n* = 55) before discharge to determine if they had a stroke. Asymptomatic cerebral emboli were defined as cerebral lesions showing radiological evidence of focal ischaemia in the diffusion-weighted imaging of MRI but did not result in acute symptoms.

In the present study, the primary outcome measure was optimal procedural success, which was defined as a maximal LVOT gradient (after provocation) <30 mmHg and MR ≤grade 1+ without mortality at 6 months after the procedure. The secondary outcome measures were a composite of major adverse events, including 30-day mortality, iatrogenic ventricular septal perforation, LV apical tear, median sternotomy conversion, iatrogenic valvular injury, instrument-related embolization or stroke. Patients were regularly followed up at 1, 3 and 6 months after surgery, and then every 6 months thereafter. Follow‐up was performed via subsequent clinic visits and telephone calls. Follow-up began on the first day after surgery and ended when the patient died for any cause or by September 2023. No patients were lost to follow-up in this study.

### Beating-heart myectomy device

**Figure 1:**
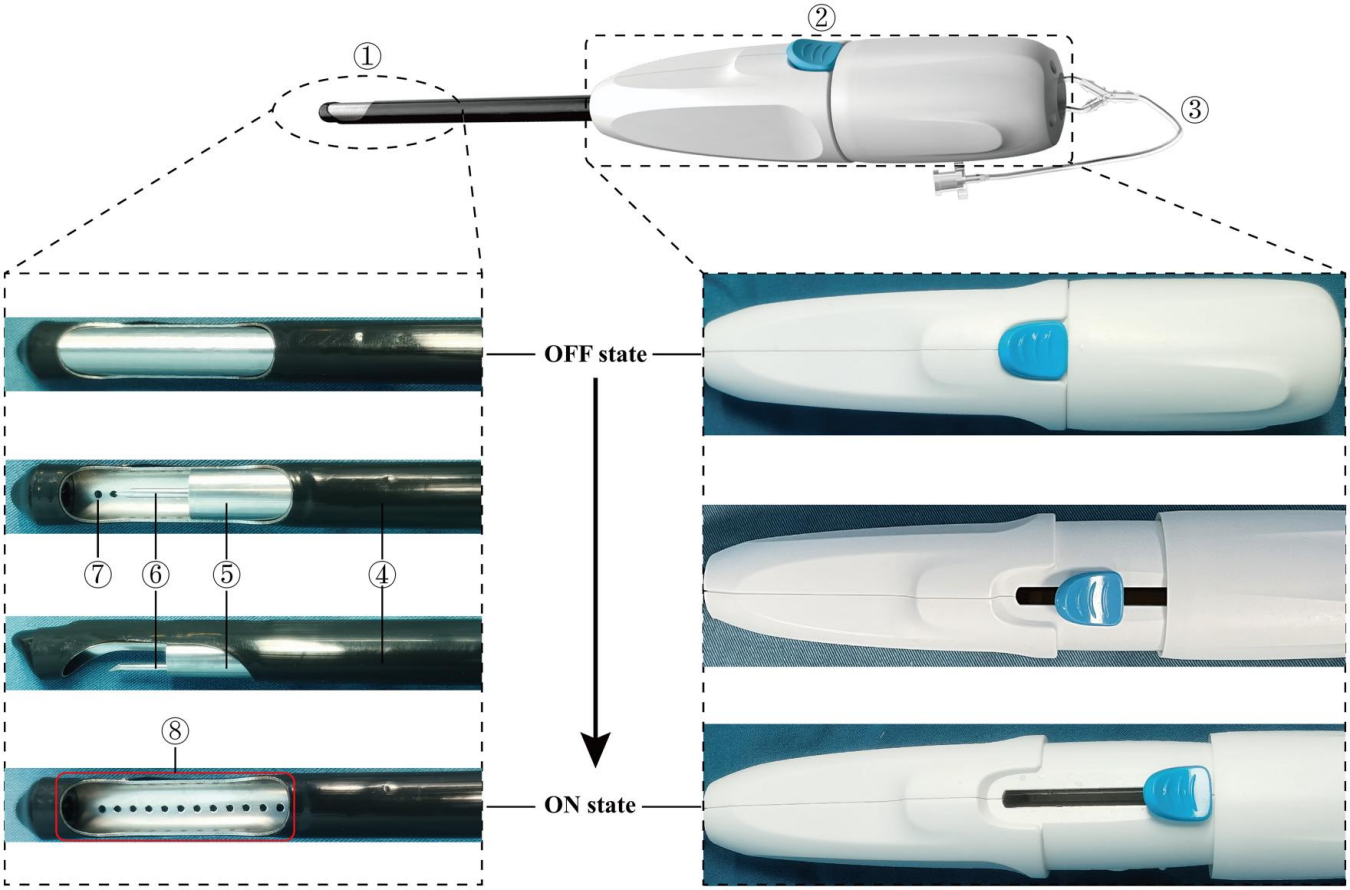
Construction of the beating-heart myectomy device and an enlarged view of the resection window and the actions between the OFF and ON state of the device.①, bullet-headed resection tube; ②, multifunctional handle; ③, catheter; ④, sleeve tube; ⑤, tubular blade; ⑥, paraxial puncture needle; ⑦, multi-porous tunnel; ⑧, resection window.

The BMD was formed of a bullet-headed resection tube, a multifunctional handle and a catheter connecting the chambers of the device (Fig. 1). The resection tube, coated with polyurethane foam to eliminate acoustic artefacts under the surveillance of echocardiography, consisted of an outer-layer sleeve tube, a tubular blade, a piercing needle and a multi-porous tunnel. The resection window on the side wall of the resection tube was sealed by the tubular blade when the device was in the OFF state. For individualized myocardial resection, a series of BMDs were modelled, including the following dimensions (length–diameter in mm): 11–30, 11–40, 13–30 and 13–40.

Before entering into the left ventricle, we carefully emptied the air from the BMD in the OFF state with normal saline through the catheter. After precise localization under transoesophageal echocardiography, the device was switched to the ON state by retracting the piercing needle and the tubular blade. The catheter was then connected to a vacuum aspirator to create a negative pressure, allowing the target myocardium to be easily pulled into the resection window. The piercing needle and tubular blade were advanced sequentially to fix and excise the target myocardium, and the device was finally returned to the OFF state. Finally, the BMD with the resected myocardium was slowly withdrawn from the left ventricle, thereby completing 1 resection.

### Procedures of transapical beating-heart septal myectomy

**Figure 2:**
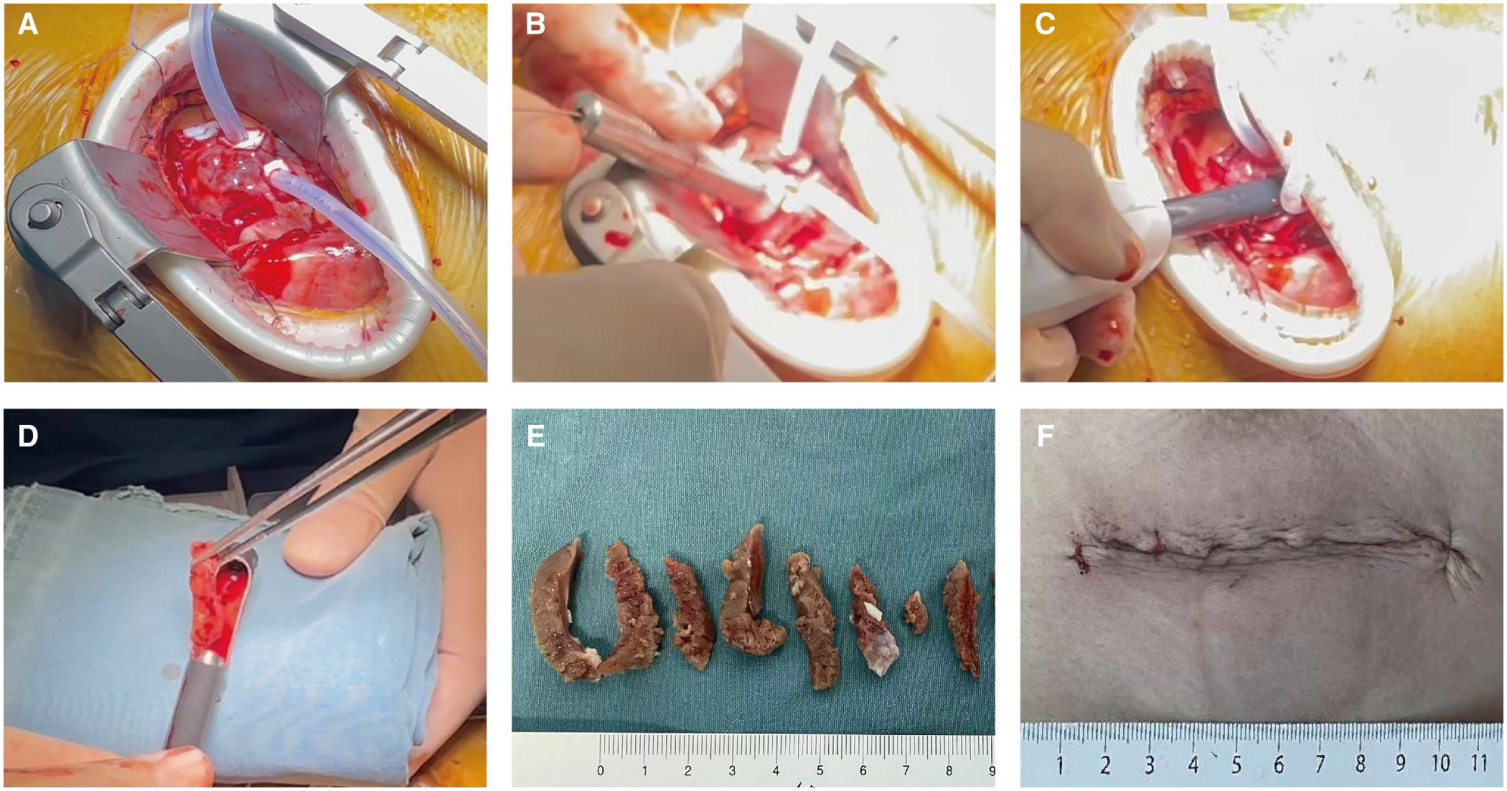
Intraoperative photographs show the transapical beating-heart septal myectomy procedure for patients with latent obstruction. The surgery is performed through the left fifth intercostal. (**A**) After the suspension of the pericardium, two-circumferential purse sutures with Teflon felt pledgets were performed in the avascular area of the cardiac apex. (**B** and **C**) After puncturing and dilating the apical incision, beating-heart myectomy device was introduced into the left ventricular cavity under the surveillance of intraoperative transoesophageal echocardiography. (**D**) The hypertrophied muscle was resected from the interventricular septum using the beating-heart myectomy device. (**E**) The surgical specimen of excised tissue is shown. (**F**) The apical and intercostal incisions were then closed.

**Figure 3:**
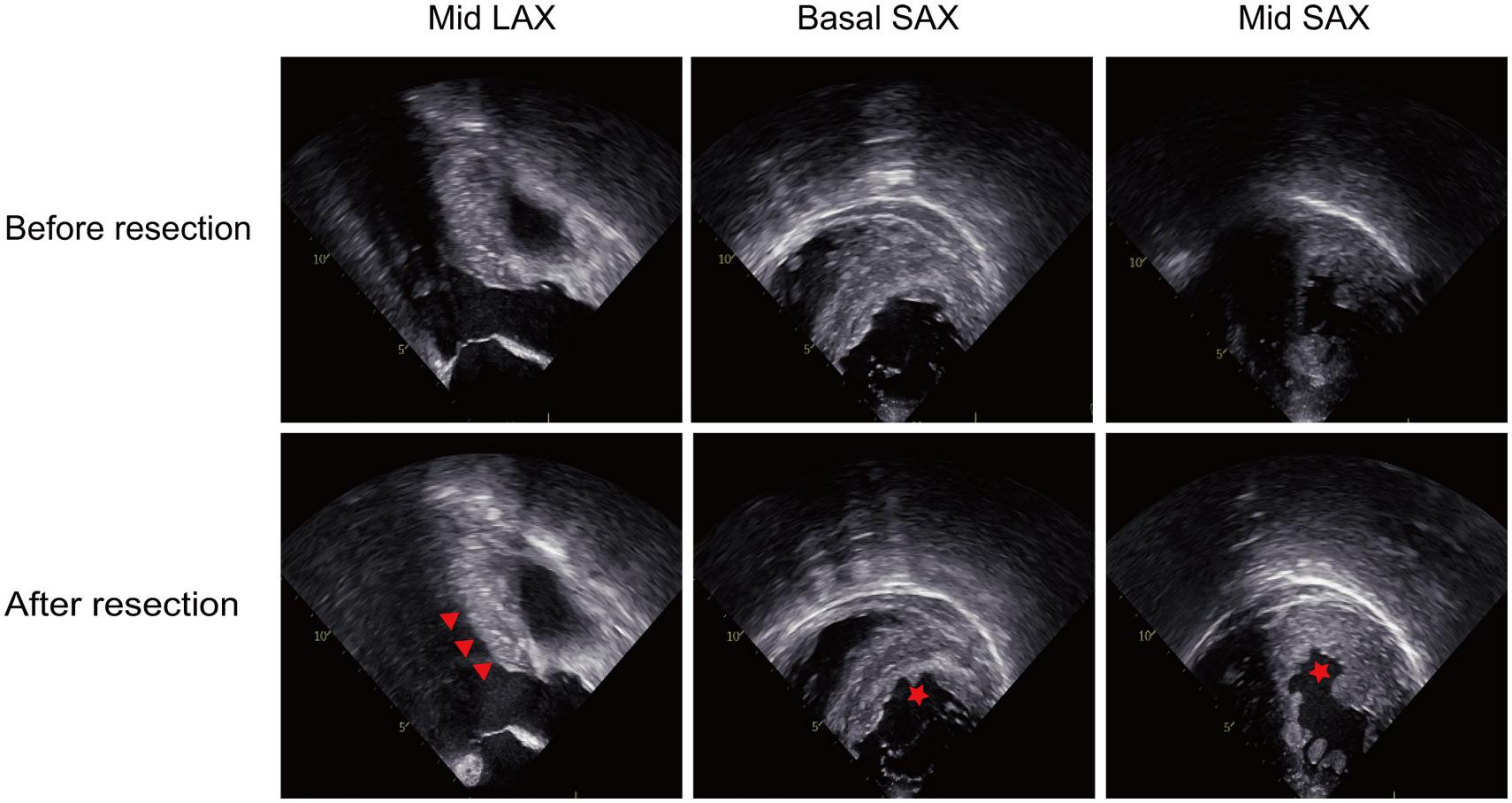
Intraoperative transoesophageal echocardiography images before and after resection. The scar created by transapical beating-heart septal myectomy is marked by red arrowheads in LAX or red pentagram in SAX. LAX: long-axis view; SAX: short-axis view.

The surgical details for the TA-BSM procedure have been previously published [7]. The procedure was performed under general anaesthesia through a left intercostal incision. The procedure began with the cardiac apex delivered anteriorly, and purse-string sutures with Teflon felt pledgets were placed in the avascular area of the apex and were fixed with snares to provide hemostasis and LV entrance for the BMD (Fig. 2) [15]. After puncturing and enlarging the apical incision, BMD was introduced into the LVOT under the guidance of intraoperative TEE. The location of the BMD was three-dimensionally determined by TEE, including the depth of the BMD tip determined in a mid-oesophagus long-axis view and the orientation of the BMD resection window in a transgastric short-axis view. After careful and accurate positioning, the resection was first performed in the basal anterior septum, locating 5–10 mm beneath the orifice of the right coronary artery in the long-axis view, and to the midpoint of the septum in the short-axis view. The morphological and haemodynamic characteristics were evaluated after each resection, including the thickness of the septum, the LVOT gradient and the MR grade. Subsequent resections were performed under the surveillance of real-time TEE. After completing the whole resections, a provocation test by isoproterenol infusion was performed to determine whether additional resections were necessary (Fig. 3). A repeat procedure was immediately performed if there was residual obstruction (a provoked gradient ≥30 mmHg). Finally, the apical puncture was closed using the purse strings. The representative procedure of TA-BSM was shown in Video 1.

### Statistics

The continuous variables were expressed as the median and interquartile range, and categorical data were expressed as counts and percentages. Group differences on the demographic variables and clinical data were analysed by Mann–Whitney tests for continuous variables and by the chi-squared test or Fisher’s exact test (the expected frequency <5) for categorical data. Within-group comparisons before and after surgery were performed using Wilcoxon’s signed rank test for continuous variables or the McNemar test for categorical data. Propensity score matching was performed of latent age, sex and body mass index. Matching was performed using the nearest neighbour method assigning patients with latent obstruction and resting obstruction in a 1:1 manner with a 0.02 calliper width.

The Kaplan–Meier method was used to calculate survival free from all-cause death. The follow-up time was calculated by censoring patients at the time of the event (death) and counting only the last follow-up time as an event from patients without an event in a Kaplan–Meier analysis (so-called inverse Kaplan–Meier) [16]. A two-sided α of <0.05 was considered statistically significant. Statistical analyses were done using the SPSS software (version 26.0).

## RESULTS

### Baseline patient characteristics

From April 2022 to February 2023, 135 symptomatic HCM patients were screened, of whom 15 were excluded due to primary mitral valve disease (*n* = 2), apical HCM (*n* = 12) and decompensated hypertrophic obstructive cardiomyopathy (*n* = 1). Therefore, a total of 120 patients were included in this study, 33 with latent obstruction and 87 with resting obstruction (Supplementary Material, Fig. S1).

In the overall cohort before propensity matching, despite the minimal obstruction at rest, patients with latent obstruction had a similar extent of physical limitation as those with resting obstruction (Table 1). The New York Heart Association (NYHA) functional classes were comparable between the 2 groups. In addition, there were no important differences in other clinical characteristics, including age, symptoms, comorbidities and medical history.

**Table 1:** Baseline patients characteristics

Variable	Latent obstruction (*n* = 33)	Resting obstruction (*n* = 87)	*P*-value
Age (years)	46 (38–56)	53 (41–60)	0.153*
Male	25 (75.8%)	57 (65.5%)	0.282^†^
Body mass index (kg/m^2^)	24.5 (20.7–28.0)	25.1 (21.6–28.0)	0.911*
Active smoker	11 (33.3%)	27 (31.0%)	0.809^†^
Family history of HCM	5 (15.2%)	9 (10.3%)	0.527^‡^
Prior septal reduction	1 (3.3%)	5 (5.7%)	0.547^†^
Septal myectomy	1 (3.3%)	2 (2.2%)	>0.999^‡^
Alcohol septal ablation	0	3 (3.4%)	>0.999^‡^
Prior PCI	2 (6.1%)	7 (8.0%)	>0.999^‡^
Prior stroke	5 (15.2%)	3 (3.4%)	0.059^‡^
Permanent pacemaker or ICD	2 (9.5%)	2 (2.2%)	0.164^‡^
NYHA class			0.378^†^
II	17 (51.5%)	33 (37.9%)	
III	14 (42.4%)	49 (56.3%)	
IV	2 (6.1%)	5 (5.7%)	
NT-pro BNP (pg/ml)	608.5 (222.8–2463.0)	1026.5 (477.3–2384.5)	0.163*
Clinical presentation			
Chest pain	21 (63.6%)	48 (55.2%)	0.402^†^
Dyspnoea	22 (66.7%)	60 (69.0%)	0.809^†^
Amaurosis	8 (24.2%)	38 (43.7%)	0.051^†^
Syncope	4 (12.1%)	21 (24.1%)	0.148^†^
Palpitation	22 (66.7%)	57 (65.5%)	0.906^†^
Comorbidities			
Hypertension	17 (51.5%)	30 (34.5%)	0.088^†^
Diabetes mellitus	5 (15.2%)	7 (8.0%)	0.307^‡^
Coronary artery atherosclerosis	13 (39.4%)	30 (34.5%)	0.616^†^
Medical therapy			
Beta‐blockers	23 (69.7%)	63 (72.4%)	0.768^†^
Calcium‐channel blockers	16 (48.5%)	33 (37.9%)	0.294^†^

Values are presented as numbers (percentages) or median (interquartile range) when appropriate.

* *P*-values were calculated by means of the Mann–Whitney test.

† *P*-values were calculated by means of the chi-squared test.

‡ *P*values were calculated by means of the Fisher’s exact test (the expected frequency <5).

HCM: hypertrophic myocardiopathy; ICD: implantable cardioverter defibrillator; NT-pro BNP: N-terminal pro-brain natriuretic peptide; NYHA: New York Heart Association; PCI: percutaneous coronary intervention.

There were marked differences in ventricular morphology and haemodynamics comparing patients with latent and resting obstruction. As shown in Table 2, patients with latent obstruction had less severe septal hypertrophy than patients with resting obstruction, as reflected in lower basal septal thickness (20 mm [16, 23] vs 22 mm [18, 25], *P* = 0.029). The left atrial chamber size was larger in patients with resting obstruction (*P* = 0.027). Patients with latent obstruction had a lower ratio of early ventricular filling to early diastolic mitral annular velocity (13 [12, 18] vs 18 [13, 24], *P* = 0.004). The proportion of MR ≥grade 2+ was significantly lower in patients with latent obstruction compared with those with resting obstruction (63.6% vs 90.8%; *P* < 0.001). In addition, patients with latent obstruction were more likely to have mitral subvalvular anomalies (30.3% vs 6.9%, *P* = 0.003), including papillary muscle anomalies (12.1% vs 2.3%, *P* = 0.048) and false tendon (18.2% vs 4.6%, *P* = 0.016).

**Table 2:** Preoperative echocardiographic, electrocardiographic and cardiac magnetic resonance data

Variable	Latent obstruction (*n* = 33)	Resting obstruction (*n* = 87)	*P*-value
Echocardiographic findings			
Left atrium diameter (mm)	41 (37–45)	44 (39–48)	0.027*
LV EDD (mm)	46 (42–48)	46 (42–49)	0.534*
Basal septum thickness (mm)	20 (16–23)	22 (18–25)	0.029*
Midventricular wall thickness (mm)	25 (16–30)	20 (17–24)	0.217*
LV EF (%)	70 (66–73)	69 (65–73)	0.491*
Resting gradients (mmHg)	20 (11–26)	88 (64–107)	<0.001*
Maximum gradients^a^ (mmHg)	77 (60–101)	88 (64–107)	0.448*
Mitral regurgitation ≥grade 2+	21 (63.6%)	79 (90.8%)	<0.001^†^
Systolic anterior motion	27 (81.8%)	78 (89.7%)	0.395^†^
Variants of HCM			0.129^†^
Basal	8 (24.2%)	38 (43.7%)	
Mid-ventricular	17 (51.5%)	36 (41.4%)	
Diffuse	8 (24.2%)	13 (14.9%)	
Mitral sub‐valvular abnormities	10 (30.3%)	6 (6.9%)	0.003^†^
Papillary muscle anomalies	4 (12.1%)	2 (2.3%)	0.048^‡^
False tendon	6 (18.2%)	4 (4.6%)	0.026^‡^
*E*/*A*	1.1 (0.8–1.6)	1.0 (0.8–1.6)	0.490*
*E*/*e*′	13 (12–18)	18 (13–24)	0.004*
Apical outpouching	3 (9.1%)	5 (5.7%)	0.806^‡^
Electrocardiographic findings			
Right bundle branch block	3 (9.1%)	3 (3.4%)	0.344^‡^
Left bundle branch block	2 (2.3%)	1 (3.0%)	>0.999^‡^
Atrial fibrillation	1 (3.0%)	7 (8.0%)	0.443^‡^
NVST	11 (33.3%)	15 (17.2%)	0.056^†^

Values are presented as numbers (percentages) or median (interquartile range) when appropriate.

a Values (with provocation) for the latent group but values (at rest) for the non-latent group.

* *P*-values were calculated by means of the Mann–Whitney test.

† *P*-values were calculated by means of the chi-squared test.

‡ *P*-values were calculated by means of the Fisher’s exact test (the expected frequency < 5).

EDD: end-diastolic dimension; EF: ejection fraction; HCM: hypertrophic cardiomyopathy; LV: left ventricle; NVST: non-sustained ventricular tachycardia.

During preoperative TTE examinations, provoked pressure gradients in the latent obstruction group were measured with the Valsalva manoeuvre (*n* = 4), treadmill test (*n* = 27), the administration of isoproterenol (*n* = 1) or repetitive squat-to-stand (*n* = 1). As shown in Supplementary Material, Fig. S2, the LVOT gradient significantly increased from 20 (11–26) mmHg to 77 (60–101) mmHg (*P* < 0.001) after provocation in patients with latent obstruction. Comparatively, patients in the latent group had significantly lower resting LVOT gradients.

In the propensity-matched cohort, the differences between 2 groups in demographic characteristics, comorbidities and imaging data were similar to the overall cohort before propensity matching (Supplementary Material, Tables S1 and S2).

### Perioperative data

In the overall cohort before propensity matching, only 8 patients required red blood cell transfusion (1 with latent obstruction and 7 with resting obstruction) (Table 2). There were no inter-group differences in the weight of resected myocardium, intensive care unit stay or new-onset left bundle branch block. The median duration of mechanical ventilation after surgery was significantly lower in patients with latent obstruction (2.9 vs 4.3 h, *P* = 0.010). Among patients without baseline right bundle branch block, 1 in the latent obstruction group developed atrioventricular block requiring permanent pacing. This patient had delayed iatrogenic ventricular septal perforation 5 h after the operation and received emergent open-heart surgery to repair the perforation. This patient received permanent pacemaker implantation due to atrioventricular block after the repair of the perforation. However, normal conduction resumed without pacemaker dependence 2 months later.

In the overall cohort before propensity matching, the composite of major adverse events occurred in 4 patients, including 2 (6.1%) in the latent obstruction group and 2 (2.3%) in the resting obstruction group (*P* = 0.649; Table 3). In the latent obstruction group, 1 patient underwent repair of delayed iatrogenic ventricular septal perforation 5 h after the TA-BSM, and the other converted to median sternotomy due to the mitral valve injury and underwent mitral mechanical valve replacement. In the resting obstruction group, 1 patient developed an LV apical tear at the end of the TA-BSM procedure and needed to repair the tear via median sternotomy, with the use of the CPB. With the presence of multiple comorbidities and right heart failure before surgery, another patient exhibited a morbid status after the surgery and died on postoperative day 10 for multi-organ failure unrelated to the instrument. No one had instrument-related embolization in this study. In addition, there was no perioperative stroke confirmed by the cranial computed tomography or MRI before discharge. Among 55 patients receiving cranial MRI, asymptomatic cerebral emboli were detected in 5 patients (1 in the latent group and 4 in the resting group). The lesions of cerebral emboli completely disappeared in 3 patients 1 month after surgery, in 1 patient 3 months after surgery and in the last 1 patient 6 months after surgery. In the propensity-matched cohort, there were no significant differences in patients’ perioperative data or clinical adverse events (Supplementary Material, Table S3).

**Table 3:** Transapical beating-heart septal myectomy procedure-associated perioperative data and clinical events

Variable	Latent obstruction (*n* = 33)	Resting obstruction (*n* = 87)	*P*-value
Duration of surgery (h)	2.8 (2.4–3.3)	2.8 (2.3–3.6)	0.621*
Weight of resected myocardium (g)	6.4 (3.0–8.2)	5.6 (3.7–9.1)	0.859*
Duration of ventilation (h)	3.0 (2.4–4.4)	4.6 (3.2–6.5)	0.010*
ICU stay (h)	21.5 (18.4–34.8)	22.2 (19.8–41.9)	0.468*
Red blood cell transfusion	1 (3.0%)	7 (8.0%)	0.443^‡^
New-onset complete left bundle branch block	14 (42.4%)	38 (43.7%)	0.901^†^
New-onset atrial fibrillation	1 (3.0%)	2 (2.3%)	>0.999^‡^
New atrioventricular block	3 (9.1%)	2 (2.3%)	0.127^‡^
New atrioventricular block without baseline RBBB^a^	1 (3.3%)	0	0.263^‡^
Asymptomatic cerebral emboli^b^	1 (7.1%)	4 (9.7%)	>0.999^‡^
Major adverse events	2 (6.1%)	2 (2.3%)	0.303^‡^
Iatrogenic ventricular septal perforation	1 (3.0%)	0	0.275^‡^
Left ventricular apical tear	0	1 (1.1%)	>0.999^‡^
Median sternotomy conversion	1 (3.0%)	1 (1.1%)	0.476^‡^
Iatrogenic valvular injury	1 (3.0%)	0	0.275^‡^
Stroke^c^	0	0	>0.999^‡^
30-Day mortality	0	1 (1.1%)	>0.999^‡^

Values are presented as numbers (percentages) or median (interquartile range).

* *P*-values were calculated by means of the Mann–Whitney test.

† *P*-values were calculated by means of the chi-squared test.

‡ *P*-values were calculated by means of the Fisher’s exact test (the expected frequency <5).

a New atrioventricular block was detected in 1 (3.3%) of 30 patients without baseline RBBB in the latent obstruction group.

b Among 55 patients receiving cranial magnetic resonance, asymptomatic cerebral emboli were detected in 1 (7.1%) of 14 patients with preoperative latent obstruction and 4 (9.4%) of 41 patients with preoperative resting obstruction.

c Stroke was detected by the cranial computed tomography or magnetic resonance imaging before discharge.

ICU: intensive care unit; RBBB: right bundle branch block.

### Follow-up results

The representative morphological alternations of TTE and CMR of patients with latent and resting obstruction are shown in Supplementary Material, Figs S3 and S4. In the overall cohort before propensity matching, over a median follow-up of 10.1 (7.4, 13.4) months, the overall survival rate was 99.2% with 1 death (10 days after surgery) in the resting obstruction group (Supplementary Material, Fig. S5). No rehospitalization for heart failure was observed in both groups.

**Figure 4:**
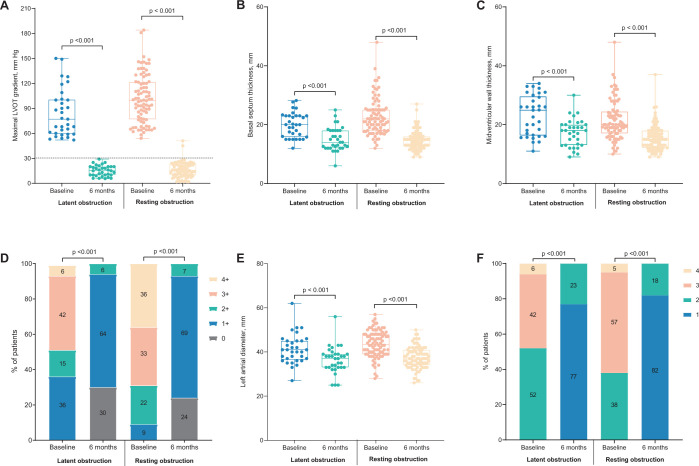
Clinical parameters at baseline and last follow-up in the total cohort. (**A**) Maximal left ventricular outflow tract gradient after provocation; (**B**) basal septal thickness; (**C**) midventricular wall thickness; (**D**) mitral regurgitation grade; (**E**) left atrial diameter; (**F**) New York Heart Association functional status.

The primary outcome, optimal procedural success, was achieved in 31 (93.9%) of 33 patients with latent obstruction and 80 (92.0%) of 87 patients with resting obstruction at 6 months after the procedure (Table 3). Follow‐up data are presented in Fig. 4 and Table 4, (i) the maximum LVOT gradients after provocation decreased to 15 (10–20) mmHg in the latent obstruction group and to 16 (10–23) mmHg in the resting obstruction group (*P* = 0.656), both of which were significantly lower than the preoperative values (both *P*-values <0.001); no patients had residual LVOT obstruction in the latent obstruction group and only 2 in the resting obstruction group (*P* > 0.999); (ii) the basal septum thickness and midventricular wall thickness were significantly smaller than that preoperatively in both 2 groups; (iii) patients in either group had significant improvements in MR severity (both *P* < 0.001); MR ≥grade 2+ was observed in 2 patients (6.1%) from the latent obstruction group and 6 (7.0%) from the resting obstruction group; there were no cases of moderate to severe or severe MR at follow-up; (iv) the left atrial diameter and the proportion of *E*/*e′* ≥15 was significantly decreased in comparison to the preoperative values, reflecting the improvement of LV diastolic function; and (v) the NYHA functional class, derived from patient survey responses, was also improved after the TA-BSM procedure and no patients were in the status of NYHA III or IV. In the propensity-matched cohort, the optimal procedural success and improvement in cardiac morphology and function in both 2 groups were similar to the overall cohort before propensity matching (Supplementary Material, Table S4).

**Table 4:** Follow-up data at 6 months after surgery

Variable	Latent obstruction (*n* = 33)	Resting obstruction (*n* = 86)	*P*-value
Optimal procedural success^a^	31 (93.9%)	80 (92.0%)	>0.999^‡^
NYHA class III or IV			
Baseline	16 (48.5%)	53 (61.6%)	0.193^†^
Last follow‐up	0	0	>0.999^‡^
Maximum LVOT gradient (mmHg)			
Baseline	77 (60–101)	87 (64–107)	0.555*
Last follow‐up	15 (10–20)	16 (10–23)	0.656*
Maximum LVOT gradient ≥30 mmHg			
Baseline	33 (100%)	86 (100%)	>0.999^‡^
Last follow‐up	0	2 (2.3%)	>0.999^‡^
Basal septum thickness (mm)			
Baseline	20 (16–23)	22 (18–25)	0.026*
Last follow‐up	14 (12–18)	15 (12–16)	0.710*
Midventricular wall thickness (mm)			
Baseline	25 (16–30)	20 (17–24)	0.227*
Last follow‐up	18 (13–20)	15 (12–18)	0.031*
Mitral regurgitation ≥ grade 2+			
Baseline	21 (63.6%)	78 (90.7%)	<0.001^†^
Last follow‐up	2 (6.1%)	6 (7.0%)	>0.999^‡^
Left atrial diameter (mm)			
Baseline	41 (37–45)	44 (39–48)	0.032*
Last follow‐up	37 (33–39)	37 (34–41)	0.337*
*E*/*e*′ ≥15			
Baseline	14 (42.4%)	56 (65.1%)	0.024*
Last follow‐up	9 (27.3%)	39 (45.3%)	0.072*

Values are presented as numbers (percentages) or median (interquartile range) when appropriate.

* *P*-values were calculated by means of the Mann–Whitney test.

† *P*-values were calculated by means of the chi-squared test.

‡ *P*-values were calculated by means of the Fisher’s exact test (the expected frequency <5).

a Optimal procedural success was achieved in 31 (93.9%) of 33 patients with latent obstruction and 80 (92.0%) of 87 patients with resting obstruction.

LVOT: left ventricular outflow tract; NYHA: New York Heart Association.

## DISCUSSION

This study showed that TA-BSM was an effective procedure to relieve LVOTO for patients with latent obstruction and resulted in 93.9% of optimal procedural success. In addition, there were no early (<30 days) and late deaths in patients with latent obstruction during a median follow-up of 11.6 (7.7, 13.8) months. Important findings from our study comparing the TA-BSM procedure between the latent obstruction and resting obstruction groups were that the overall survival did not differ. Further, the degree of symptomatic improvement and relief of LVOT gradient were similar between the 2 groups after TA-BSM.

Previous studies have reported that about 70–75% of the HCM patients had latent or resting obstruction [3, 17]. Some studies indicated that patients with latent obstruction generally had a better survival than those with resting obstruction or no obstruction [18, 19]. In addition, Schaff *et al.* [20] reported that symptomatic patients with latent obstruction benefited from SM with excellent survival and symptom relief. Although latent obstruction is present in almost the same number of patients as those with resting obstruction [3], in a recent study from the Mayo Clinic, the number of patients undergoing SM for latent obstruction was only half that of patients with resting obstruction [21]. In the present study, the proportion of patients with latent obstruction was approximately two-fifths that of patients with resting obstruction, indicating that patients with latent obstruction might be less likely to receive surgical myectomy.

Despite the large difference in resting LVOT gradients, patients with latent obstruction had similarly reduced preoperative functional capacity, as indicated by the NYHA class. A previous study stated that latent obstruction may represent an earlier and/or a milder morphological subtype of obstructive HCM [21]. In the present study, it is interesting to note that patients with latent obstruction had lower basal septal thickness and a significantly higher prevalence of mitral subvalvular anomalies. It was difficult to explain that mitral subvalvular abnormalities tended to decrease in the progression from latent obstruction to resting obstruction. Therefore, latent obstruction may be an isolated type of obstructive HCM, characterized by drug refractory symptoms, predominant basal hypertrophy and mitral subvalvular anomalies. The primary cause of exertional symptoms may be difficult to determine in patients with latent obstruction, and clinicians may conclude that their physical limitation results from myocardial stiffness and diastolic dysfunction [21]. Thus, in patients with less marked basal hypertrophy who had LVOT gradient <30 mmHg at rest and exertional symptoms, provocation manoeuvres such as the Valsalva, treadmill test or stand-to-squat should be considered to determine whether they had latent obstruction.

There was no difference in surgical details between patients with latent obstruction and resting obstruction. A recent study reported that a majority of patients with latent obstruction had mitral subvalvular abnormalities and suggested that concomitant mitral subvalvular procedures during myectomy were important for relieving provoked obstruction [9]. However, concomitant mitral valve procedures have been performed only in 2.1% of obstructive HCM patients without intrinsic MV disease in the Mayo Clinic [22]. Further, in another study of 629 patients with latent obstruction, the prevalence of concomitant mitral valve procedures was comparable in patients with latent obstruction and resting obstruction [21]. In this study, 10 patients (30.3%) with latent obstruction had mitral sub‐valvular abnormalities, but none of them received concomitant mitral valve procedures. In our study, among 8 patients with baseline apical outpouching, the purse-string sutures with Teflon felt pledgets were located in the area of the apical outpouching to provide LV entrance for the BMD. After complete resections, the apical outpouching was solved after the suture was drawn tight and tied. During the follow-up, there was no apical outpouching or aneurysm related to the apical incision.

The major drawbacks of conventional SM are the limited surgical field and the lack of real-time evaluation. However, the comprehensive and broadened visualization of LV geometry is abled during TA-BSM through the echocardiography-implemented cross-sectional view. Not only morphological parameters but haemodynamic characteristics can be carefully re-evaluated after each resection. Therefore, we can accurately locate the target septal myocardium and safely perform repeated resection to adjust the extent of myectomy, thereby fully eliminating LVOTO and MR. Therefore, even by strict standards, 111 patients (31 [93.9%] with latent obstruction and 80 [92.0%] with resting obstruction) achieved optimal procedural success, which was defined as a maximal LVOT gradient (after provocation) <30 mmHg and MR ≤grade 1+ without mortality at 6 months after surgery. Furthermore, surgical trauma by median sternotomy and CPB-induced injury can be minimized, resulting in short median durations of ventilation (4.1 h) and intensive care unit stay (21.8 h) and less need for blood transfusion (8 patients, 7.3%).

Fifty-two (43.3%) of 120 patients developed left bundle branch block after TA-BSM, which was similar to the large series of 2,482 patients with obstructive HCM [23]. Among 114 patients without baseline right bundle branch block, 1 (0.9%) developed atrioventricular block requiring permanent pacing, which was comparable to other large series [21, 23]. In addition, 1 resumed normal conduction without pacemaker dependence 2 months after surgery. In our cohort, the incidence of new-onset atrial fibrillation occurred in 3 (2.5%) patients, which seemed lower than 11–13% of prior studies [24, 25]. This favourable impact on atrial fibrillation was likely explained by the promising clinical outcomes of TA-BSM, including the reduction in maximum LVOT gradient, MR severity and left atrial size [26, 27]. In addition, it was reported that the minimally invasive approach for cardiac surgery reduced postoperative atrial fibrillation when compared with median sternotomy [28]. The reduction of atrial fibrillation may be due to decreased trauma leading to a reduction in inflammation. However, further studies are needed to determine the exact mechanism.

Although numerical differences were observed, the incidence of major adverse events was not statistically significant (6.1% vs 2.3%, *P* = 0.649), which included a composite of 30-day mortality, ventricular septal perforation, LV apical tear, median sternotomy conversion, iatrogenic valvular injury, instrument-related embolization or stroke. In this study, the differences in overall 30-day survival were not statistically significant (100% vs 98.9%), which was similar to previous studies [8, 21]. Ventricular septal perforation occurred in 1 (0.8%) patient with a preoperatively maximal septal thickness of 15.2 mm and with a postoperatively minimum residual septal thickness of 5.9 mm. Therefore, the resection should be performed cautiously in patients with mild septal hypertrophy. In a recent study of 583 patients receiving SM, 9 (1.5%) had the occurrence of ventricular septal perforation [29], which was a little higher than our study. In addition, LV apical tear occurred in 1 (0.8%) patient at the end of the TA-BSM procedure, the rate of which was similar to previous studies [9, 29]. We successfully managed this rare complication via bailout surgery to repair the tear via median sternotomy, with the use of CPB. Unlike transaortic SM may result in aortic valve injury [9], mitral valve prolapses occurred in 1 (0.8%) patient attributed to severed tendon cords. The patient subsequently conversed to median sternotomy and underwent mechanical mitral valve replacement.

In this study, TA-BSM showed excellent surgical results and follow-up measurement demonstrated a dramatic relief of provoked LVOT gradient. Relief of LVOT obstruction relieves systolic anterior motion-associated mitral valve regurgitation [22]. In our experience, MR always gradually vanished following sequential resections without concomitant mitral valve procedure and a majority (92.5%, 111/120) of patients with pre-existing MR showed MR ≤grade 1+ at their 6-month follow-up assessment. It is suggested that adequate septal reduction is sufficient to alleviate MR and concomitant mitral valve surgery is unnecessary unless in patients with native mitral valve disease. Moreover, in the presence of decreased septum thickness and left atrial diameter after TA-BSM, reverse remodelling of the left ventricle and atrium may also contribute to improved diastolic function [26, 30]. In the present study, patients with latent obstruction had significant improvement in symptoms following the TA-BSM confirming the importance of LVOT obstruction in functional impairment. The good survival and improved symptoms after surgery are strong support for TA-BSM in HCM patients with latent obstruction and resting obstruction.

### Limitations

The present study had some potential limitations. First, this was a single-centre study and the sample size was relatively small, which may have led to selection bias and influenced the generalizability of the results. Furthermore, uncontrolled nature made any causal inference impossible and kept the study at an exploratory level only. Second, the TA-BSM procedure was not suitable for patients with comorbidities requiring open-heart surgery, such as primary valvular disease and coronary artery disease. In addition, high-quality images of transoesophageal echocardiography were needed to precisely locate and resect the hypertrophied myocardium during the procedure, which requires the close cooperation of the sonologist. Third, the assessment of postoperative functional status in our study was based on patients’ self-reporting and was, therefore, subjective; none of them underwent cardiopulmonary exercise testing before and after TA-BSM to confirm their functional capacity. Finally, only a few patients have undergone gene analysis, and more studies are needed to identify the different genetic anomalies between patients with latent obstruction versus resting obstruction.

## CONCLUSIONS

TA-BSM preserved favourable gold-standard guideline desired outcomes through real-time echocardiographic-guided resection. Equipoise of outcomes for this procedure regardless of degree of resting LVOT gradients supports operative management with this approach in symptomatic patients with latent obstruction. These findings, however, remain to be confirmed in a larger series with long-term follow-up.

## Supplementary Material

ezad425_Supplementary_Data

## Data Availability

The authors confirm that the data supporting the findings of this study are available within the article and its supplementary materials.

## ABBREVIATIONS

BMD: Beating-heart myectomy device
CPB: Cardiopulmonary bypass
HCM: Hypertrophic cardiomyopathy
LV: Left ventricular
LVOT: Left ventricular outflow tract
MR: Mitral regurgitation
MRI: Magnetic resonance imaging
NYHA: New York Heart Association
SM: Septal myectomy
TA-BSM: Transapical beating-heart septal myectomy
TTE: Transthoracic echocardiography
