# Supplementary material for: Transapical beating-heart septal myectomy for hypertrophic cardiomyopathy with latent obstruction
Source: Eur J Cardiothorac Surg. 2023 Dec 19;65(1):ezad425. doi: 10.1093/ejcts/ezad425 (PMC10903174; doi:10.1093/ejcts/ezad425)
Supplement: ezad425_Supplementary_Data [file ezad425_Supplementary_Data.zip › Supplementary material.docx]

**Supplementary Figures**

Supplementary Figure 1. Flow chart for the selection of study patients.

HOCM, hypertrophic obstructive cardiomyopathy; LVOT, left ventricular outflow tract.


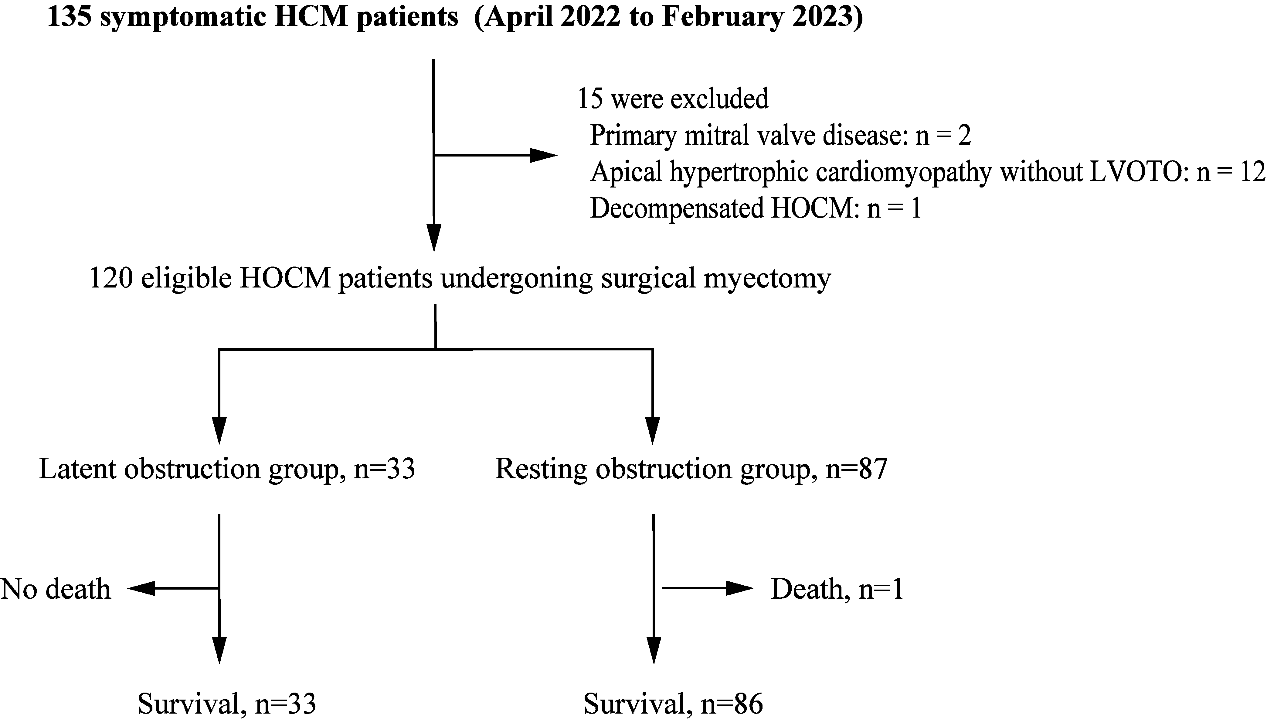


Supplementary Figure 2. Changes of instantaneous LVOT gradient with Valsalva maneuver, treadmill test, isoproterenol infusion, or repetitive squat-to-stand maneuver in patients with HCM and latent obstruction during transthoracic echocardiography.

LVOT gradient increased from 20 (11-26) mm Hg at rest to 77 (60-101) mm Hg after provocation (P< 0.001). LVOT, Left ventricular outflow tract; HCM, hypertrophic cardiomyopathy.


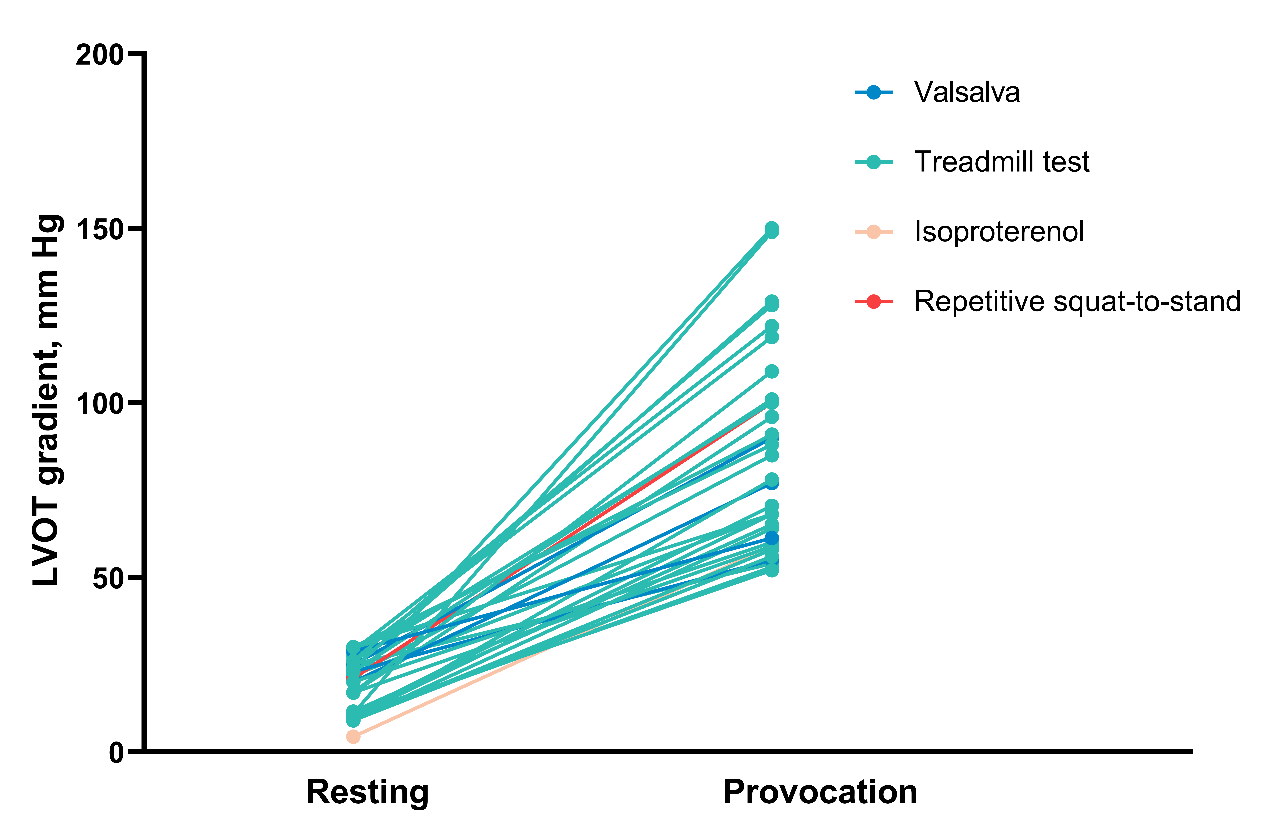


Supplementary Figure 3. Representative transthoracic echocardiography and cardiac magnetic resonance images at baseline and 4 months after TA-BSM in a 59 years old man with latent obstruction (provoked LVOTG of 100 mmHg despite of low resting LVOTG of 11 mmHg), as indicated. The scar created by TA-BSM is marked by * in SAX or black arrows in LAX.

A3C color, color Doppler under the apical three-chamber view; A3C CW, continuous wave Doppler under the apical three-chamber view; CMR, cardiac magnetic resonance; LAX, long-axis view; LVOTG, left ventricular outflow tract gradient; MR, mitral regurgitation; SAX, short-axis view; TA-BSM, transapical beating-heart septal myectomy.
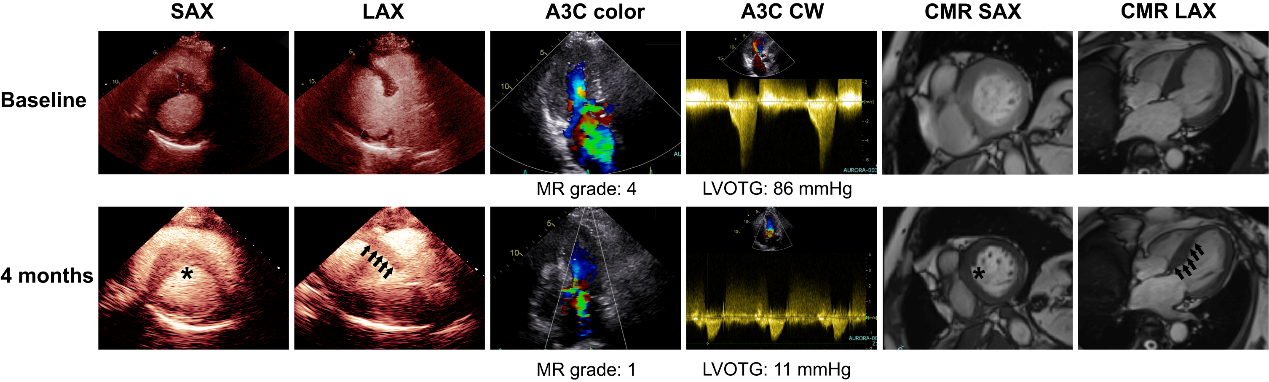


Supplementary Figure 4. Representative transthoracic echocardiographic and cardiac magnetic resonance images at baseline and 4 months after TA-BSM in a 72 years old female with resting obstruction, as indicated. The scar created by TA-BSM is marked by * in SAX or black arrows in LAX.

A3C color, color Doppler under the apical three-chamber view; A3C CW, continuous wave Doppler under the apical three-chamber view; CMR, cardiac magnetic resonance; LAX, long-axis view; LVOTG, left ventricular outflow tract gradient; MR, mitral regurgitation; SAX, short-axis view; TA-BSM, transapical beating-heart septal myectomy.


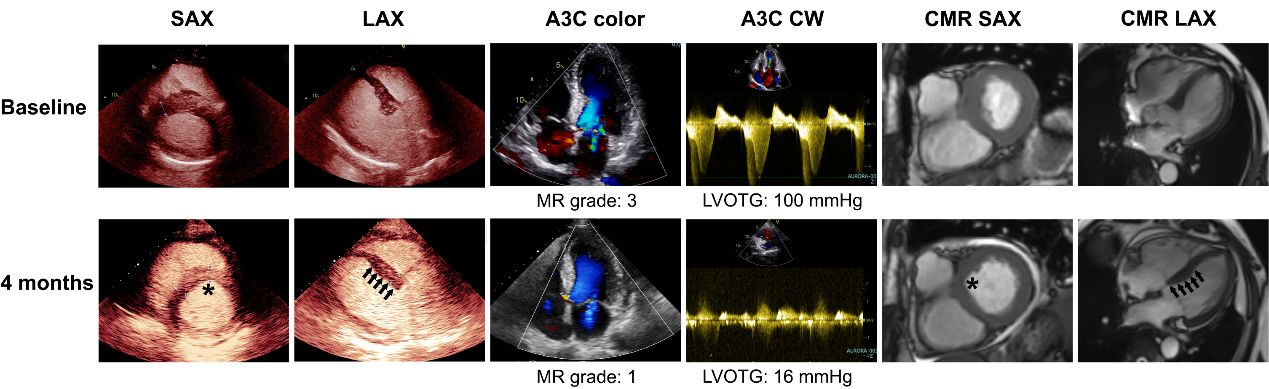


Supplementary Figure 5. Survival of patients with obstructive hypertrophic cardiomyopathy following the transapical beating-heart septal myectomy (TA-BSM) procedure stratified by latent obstruction and resting obstruction.


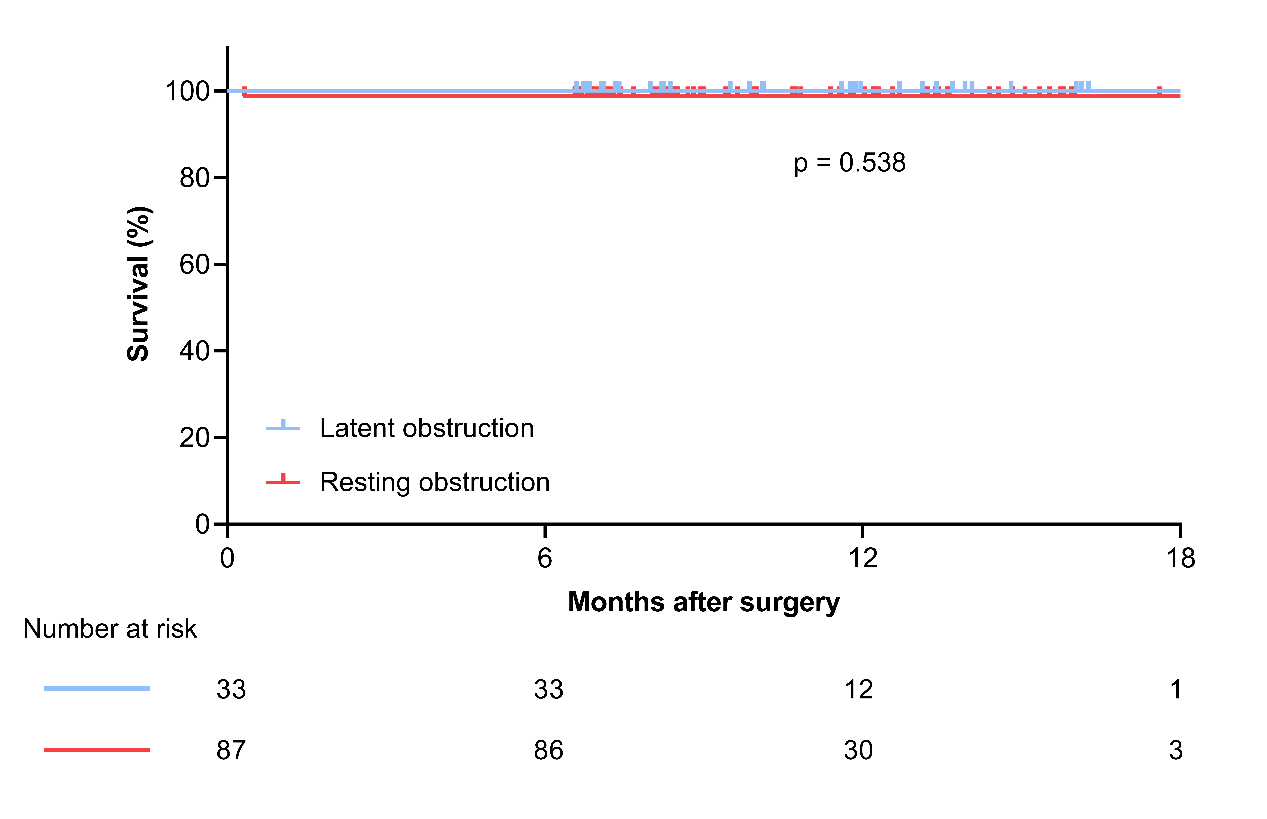


**Supplementary Tables**

Supplementary Table 1 Baseline patients’ characteristics after propensity matching.

| Variable | Latent obstruction (n=33) | Resting obstruction (n=33) | P value |
| --- | --- | --- | --- |
| Age, years | 46 (38-56) | 53 (42-59) | 0.311* |
| Male | 25 (75.8%) | 27 (81.8%) | 0.547† |
| Body mass index, kg/m^2^ | 24.5 (20.7-28.0) | 25.3 (21.9-28.6) | 0.621* |
| Active smoker | 11 (33.3%) | 13 (39.4%) | 0.609† |
| Family history of HCM | 5 (15.2%) | 3 (9.1%) | 0.708‡ |
| Prior septal reduction | 1 (3.3%) | 3 (9.1%) | 0.613‡ |
| Septal myectomy | 1 (3.3%) | 2 (6.1%) | >0.999‡ |
| Alcohol septal ablation | 0 | 1 (3.0%) | >0.999‡ |
| Prior PCI | 2 (6.1%) | 4 (12.1%) | 0.672‡ |
| Prior stroke | 5 (15.2%) | 1 (3.0%) | 0.197‡ |
| Permanent pacemaker or ICD | 2 (9.5%) | 2 (2.2%) | 0.164‡ |
| NYHA class |  |  | 0.131† |
| II | 17 (51.5%) | 9 (27.3%) |  |
| III | 14 (42.4%) | 21 (63.6%) |  |
| IV | 2 (6.1%) | 3 (9.1%) |  |
| NT-pro BNP, pg/ml | 608.5 (222.8-2463.0) | 1134.0 (573.0-2240.0) | 0.203* |
| Clinical presentation |  |  |  |
| Chest pain | 21 (63.6%) | 20 (60.6%) | 0.800† |
| Dyspnea | 22 (66.7%) | 21 (63.6%) | 0.798† |
| Amaurosis | 8 (24.2%) | 15 (45.5%) | 0.071† |
| Syncope | 4 (12.1%) | 6 (18.2%) | 0.492† |
| Palpitation | 22 (66.7%) | 21 (63.6%) | 0.796† |
| Comorbidities |  |  |  |
| Hypertension | 17 (51.5%) | 12 (36.4%) | 0.215† |
| Diabetes mellitus | 5 (15.2%) | 7 (8.0%) | 0.307‡ |
| Coronary artery atherosclerosis | 13 (39.4%) | 9 (27.3%) | 0.296† |
| Medical therapy |  |  |  |
| Beta‐blockers | 23 (69.7%) | 26 (78.8%) | 0.398† |
| Calcium‐channel blockers | 16 (48.5%) | 13 (39.4%) | 0.457† |

Note: Values are presented as numbers (percentages) or median (interquartile range) when appropriate.

* P values were calculated by means of the Mann-Whitney test.

† P values were calculated by means of the chi-squared test.

‡ P values were calculated by means of the Fisher’s exact test (the expected frequency < 5).

Abbreviations: HCM, hypertrophic myocardiopathy; ICD, implantable cardioverter defibrillator; NT-pro BNP, N-terminal pro-brain natriuretic peptide; NYHA, New York Heart Association; PCI, percutaneous coronary intervention.

Supplementary Table 2 Preoperative echocardiographic, electrocardiographic and CMR data after propensity matching.

| Variable | Latent obstruction (n = 33) | Resting obstruction (n = 33) | P value |
| --- | --- | --- | --- |
| Echocardiographic findings |  |  |  |
| Left atrium diameter, mm | 41 (37-45) | 46 (42-50) | 0.001* |
| LV EDD, mm | 46 (42-48) | 46 (42-52) | 0.375* |
| Basal septum thickness, mm | 20 (16-23) | 21 (18-25) | 0.145* |
| Midventricular wall thickness, mm | 25 (16-30) | 20 (17-24) | 0.253* |
| LV EF, % | 70 (66-73) | 69 (64-72) | 0.375* |
| Resting gradients, mm Hg | 20 (11-26) | 93 (71-111) | <0.001* |
| # Maximum gradients, mm Hg | 77 (60-101) | 93 (71-111) | 0.151* |
| Mitral regurgitation ≥ grade 2+ | 21 (63.6%) | 30 (90.9%) | 0.008† |
| Systolic anterior motion | 27 (81.8%) | 30 (90.9%) | 0.475‡ |
| Variants of HCM |  |  | 0.293† |
| Basal | 8 (24.2%) | 14 (42.4%) |  |
| Mid-ventricular | 17 (51.5%) | 13(39.4%) |  |
| Diffuse | 8 (24.2%) | 6 (18.2%) |  |
| Mitral sub‐valvular abnormities | 10 (30.3%) | 1 (3.0%) | 0.011† |
| Papillary muscle anomalies | 4 (12.1%) | 0 | 0.114‡ |
| False tendon | 6 (18.2%) | 1 (3.0%) | 0.105‡ |
| E/A | 1.1 (0.8-1.6) | 1.1 (0.8-1.7) | 0.861* |
| E/e′ | 13 (12-18) | 18 (15-26) | 0.001* |
| Apical outpouching | 3 (9.1%) | 0 | 0.238‡ |
| Electrocardiographic findings |  |  |  |
| Right bundle branch block | 3 (9.1%) | 0 | 0.238‡ |
| Left bundle branch block | 2 (2.3%) | 1 (3.0%) | >0.999‡ |
| Atrial fibrillation | 1 (3.0%) | 3 (9.1%) | 0.613‡ |
| NVST | 11 (33.3%) | 5 (15.2%) | 0.085† |

Note: Values are presented as numbers (percentages) or median (interquartile range) when appropriate.

* P values were calculated by means of the Mann-Whitney test.

† P values were calculated by means of the chi-squared test.

‡ P values were calculated by means of the Fisher’s exact test (the expected frequency < 5).

# Values (with provocation) for the latent group but values (at rest) for the non-latent group.

Abbreviations: CMR, cardiac magnetic resonance; EDD, end-diastolic dimension; EDV, end-diastolic volume; EF, ejection fraction; ESV, end-systolic volume; HCM, hypertrophic cardiomyopathy; LV, left ventricle; NVST, non-sustained ventricular tachycardia.

Supplementary Table 3 TA-BSM procedure-associated perioperative data and clinical events after propensity matching.

| Variable | Latent obstruction (n = 33) | Resting obstruction (n = 33) | P value |
| --- | --- | --- | --- |
| Duration of surgery, h | 2.8 (2.4-3.3) | 2.9 (2.4-3.8) | 0.472* |
| Weight of resected myocardium, g | 6.4 (3.0-8.2) | 5.2 (3.7-8.6) | 0.817* |
| Duration of ventilation, h | 3.0 (2.4-4.4) | 4.1 (3.1-6.4) | 0.056* |
| ICU stay, h | 21.5 (18.4-34.8) | 21.7 (19.7-27.9) | 0.720* |
| Red blood cell transfusion | 1 (3.0%) | 3 (9.1%) | 0.613‡ |
| New-onset complete left bundle branch block | 14 (42.4%) | 15 (45.5%) | 0.804† |
| New-onset atrial fibrillation | 1 (3.0%) | 1 (3.0%) | >0.999‡ |
| New atrioventricular block | 3 (9.1%) | 0 | 0.238‡ |
| New atrioventricular block without baseline RBBB^$^ | 1 (3.3%) | 0 | 0.476‡ |
| Asymptomatic cerebral emboli^&^ | 1 (7.1%) | 2 (10.5%) | >0.999‡ |
| Major adverse events | 2 (6.1%) | 2 (2.3%) | 0.492‡ |
| Iatrogenic ventricular septal perforation | 1 (3.0%) | 0 | >0.999‡ |
| Left ventricular apical tear | 0 | 0 | >0.999‡ |
| Median sternotomy conversion | 1 (3.0%) | 0 | >0.999‡ |
| Iatrogenic valvular injury | 1 (3.0%) | 0 | >0.999‡ |
| Stroke^#^ | 0 | 0 | >0.999‡ |
| 30-d mortality | 0 | 0 | >0.999‡ |

Note: Values are presented as numbers (percentages) or median (interquartile range).

* P values were calculated by means of the Mann-Whitney test.

† P values were calculated by means of the chi-squared test.

‡ P values were calculated by means of the Fisher’s exact test (the expected frequency < 5).

$ New atrioventricular block was detected in one (3.3%) of 30 patients without baseline RBBB in the latent obstruction group.

& Among 33 patients receiving cranial magnetic resonance, asymptomatic cerebral emboli were detected in one (7.1%) of 14 patients with preoperative latent obstruction and two (10.2%) of 19 patients with preoperative resting obstruction.

# Stroke was detected by the cranial computed tomography or magnetic resonance imaging before discharge.

Abbreviations: ICU, intensive care unit; RBBB, right bundle branch block; TA-BSM, transapical beating-heart septal myectomy.

Supplementary Table 4 Follow‐up data at 6 months after propensity matching.

| Variable | Latent obstruction (n = 33) | Resting obstruction (n = 33) | P value |
| --- | --- | --- | --- |
| Optimal procedural success | 31 (93.9%) | 30 (90.9%) | >0.999‡ |
| NYHA class III or IV |  |  |  |
| Baseline | 16 (48.5%) | 24 (72.7%) | 0.044† |
| Last follow‐up | 0 | 0 | >0.999‡ |
| Maximum LVOT gradient, mm Hg |  |  |  |
| Baseline | 77 (60-101) | 93 (71-111) | 0.151* |
| Last follow‐up | 15 (10-20) | 22 (15-25) | 0.656* |
| Maximum LVOT gradient ≥30 mmHg |  |  |  |
| Baseline | 33 (100%) | 33 (100%) | >0.999‡ |
| Last follow‐up | 0 | 1 (3.0%) | >0.999‡ |
| Basal septum thickness, mm |  |  |  |
| Baseline | 20 (16-23) | 21 (18-25) | 0.145* |
| Last follow‐up | 14 (12-18) | 14 (12-16) | 0.842* |
| Midventricular wall thickness, mm |  |  |  |
| Baseline | 25 (16-30) | 20 (17-24) | 0.253* |
| Last follow‐up | 18 (13-20) | 15 (13-18) | 0.145* |
| Mitral regurgitation ≥ grade 2+ |  |  |  |
| Baseline | 21 (63.6%) | 30 (90.9%) | 0.008† |
| Last follow‐up | 2 (6.1%) | 3 (9.1%) | >0.999‡ |
| Left atrial diameter, mm |  |  |  |
| Baseline | 41 (37-45) | 46 (42-50) | 0.001* |
| Last follow‐up | 37 (33-39) | 39 (35-41) | 0.114* |
| E/e′ ≥15 |  |  |  |
| Baseline | 14 (42.4%) | 25 (75.8%) | 0.006* |
| Last follow‐up | 9 (27.3%) | 13 (39.4%) | 0.296* |

Note: Values are presented as numbers (percentages) or median (interquartile range) when appropriate.

* P values were calculated by means of the Mann-Whitney test.

† P values were calculated by means of the chi-squared test.

‡ P values were calculated by means of the Fisher’s exact test (the expected frequency < 5).

Abbreviations: LVOT, left ventricular outflow tract; NYHA, New York Heart Association.
